# Complete Sequence and Characterization of Mitochondrial and Chloroplast Genome of *Navicula incerta* CACC 0356

**DOI:** 10.3390/life15010102

**Published:** 2025-01-15

**Authors:** Zhaokai Wang, Xiaoyu Wei

**Affiliations:** Technical Innovation Center for Utilization of Marine Biological Resources, Third Institute of Oceanography, Ministry of Natural Resources, Xiamen 361000, China

**Keywords:** *Navicula incerta*, mitochondrial genome, chloroplast genome, comparative analysis, repeat sequence

## Abstract

*Navicula incerta*, a marine benthic diatom, holds promise for human nutrition and health as well as for aquaculture applications. However, the scarcity of organelle genome data within the Navicula clade has impeded a comprehensive understanding and utilization of this group. Our research presents a pioneering exploration into the complete mitochondrial and chloroplast genome sequences of *N. incerta* CACC 0356, shedding light on its phylogeny and evolutionary history. The mitochondrial genome (mtDNA) spans 61,320 bp with a GC content of 29.87%, comprising one circular DNA molecule that encodes for 34 protein-coding genes, 24 tRNAs, and 34 rRNAs. Whereas, the chloroplast genome (cpDNA) is larger at 129,090 bp, encompassing 127 protein-coding genes, 30 tRNAs, and 7 rRNAs. Notably, the cpDNA of *N. incerta* is approximately 2.1 times the size of its mtDNA. Our annotation identified four genes that are partially situated in the homologous regions between the mitogenome and chloroplast genome, totaling 372 bp, which represents 0.61% of the entire mitogenome. Comparative analyses revealed that *N. incerta* CACC 0356 is closely affiliated with *Fistulifera saprophila* and *Fistulifera solaris*, both members of the Naviculaceae order. This study significantly expands the mitochondrial and chloroplast genomic resources for algae and lays a foundation for the development of genetic diversity analyses in algae.

## 1. Introduction

Diatoms, members of the class Bacillariophyceae within the phylum Bacillariophyta, are microscopic, photosynthetic algae that serve as vital primary producers within aquatic ecosystems [1,2]. Among these, *Navicula incerta* is a species that has garnered attention due to its ecological significance and potential applications in various biotechnological fields, including as a natural bait, a bioremediation agent, and a source for the production of bioactive molecules [3]. The genus *Navicula* is particularly diverse, with *N. incerta* being one of the most common species found in both planktonic and benthic habitats [4,5].

The examination of organelle genomes is of paramount importance for deciphering the phylogenetic relationships and evolutionary histories among species. The mitochondrial genomes in diatoms, while less frequently studied, can offer clues about the interactions of diatoms with their environment and their role in carbon cycling [6,7]. Meanwhile, chloroplast genomes are characterized by slow evolution, conserved genetics, and a wealth of mutation site information, making them invaluable for phylogenetic studies and species identification within the Bacillariophyceae class [8]. The importance of studying the organelle genomes of *N. incerta* is further highlighted by the fact that these genomes serve as “super barcodes”, providing enhanced resolution for discerning different species and genomic variations compared to traditional molecular markers [9,10]. For *N. incerta*, understanding its mitochondrial and chloroplast genomic architectures are essential for elucidating its evolutionary relationships, assessing its biodiversity, and potentially exploiting its biotechnological applications. Thus far, only a handful of diatom mtDNA and cpDNA have been sequenced, like *Haslea ostrearia* [11], *Thalassiosira pseudonana* [12], and *Nitzschia palea* [13]. Nevertheless, the scarcity of organelle genome data for the Navicula clade, in contrast to the more extensively studied *Chlorella* and *Parachlorella* clades, hinders a thorough comprehension of algal genome evolution.

This study presents the mtDNA and cpDNA sequences of *N. incerta* CACC 0356. The objectives of this study were to elucidate the structural characteristics of the mtDNA and cpDNA of *N. incerta* and to extend our investigation by engaging in a comparative genomic analysis across species within the Bacillariophyceae clade. This study aims to shed light on the genetic landscape and evolutionary dynamics of these important microalgae.

## 2. Materials and Methods

### 2.1. Samples, DNA Extraction and Sequencing

The algal powder of *N. incerta* was dispatched to Genepioneer Biotechnologies (Nanjing, China) for sequencing services. [14]. In our quest for high-precision full-length mtDNA sequencing, this study utilized an integrated approach, combining short-read and long-read sequencing technologies. The short-read sequencing was performed on the Illumina Novaseq 6000 platform (Illumina, San Diego, CA, USA), with paired-end reads of 150 bp in length. We utilized fastp software, version 0.20.0, available at https://github.com/OpenGene/fastp (accessed on 18 December 2023), to preprocess the raw data and obtain high-quality reads. For long-read sequencing, the Nanopore PromethION platform (Nanopore, Oxford, UK) was employed, and the resulting data were refined using the filtlong software, version 0.2.1, which can be found at https://link.zhihu.com/?target=https%3A//github.com/rrwick/Filtlong (accessed on 18 December 2023).

### 2.2. Morphological Analysis

The viable cells in the logarithmic growth phase were examined under a ZEISS Axio Imager Z2 light microscope, manufactured by Zeiss in Sliedrecht, The Netherlands, by employing a ×100 oil immersion objective lens. The microscopic images were subsequently captured using a high-resolution Zeiss Axiocam 506 color digital camera (Berlin, Germany). Algae morphology was observed using SEM. The morphology was examined with a scanning electron microscopy (SEM) (Sigma, Hitachi, Tokyo, Japan).

### 2.3. Genome Assembly and Annotation

Capitalizing on the highly conserved nature of genes in algal mtDNA, including coding sequences (CDS) and rRNA, we employed the comparison software Minimap2, version 2.1, to align the original long-read sequencing data against a reference gene sequence from plant mtDNA [15]. The fragments with significant sequence similarity, exceeding 50 base pairs in length, were identified as potential candidates. Among these, the sequences harboring a greater number of aligned genes and exhibiting a superior alignment quality—encompassing more complete core genes—were chosen as the seed sequences. Subsequently, the two sequences were compared, and those with a minimum overlap of 1 kilobase and at least 70% similarity were integrated into the seed sequence. The process was iterated to align the two sequences, thereby compiling the complete mitochondrial genome data.

We then utilized the assembly software Canu to refine the long-read sequencing data and bowtie2, version 2.3.5.1, to map the short-read sequencing data onto the corrected sequence [16]. Unicycler, version 0.4.8, was employed with default parameters to merge the above short-read sequencing data with the corrected long-read sequencing data, culminating in the acquisition of the circular *N. incerta* mtDNA.

The annotation of the mtDNA structure was conducted through the following steps: (1) the protein coding genes (PCGs) and rRNA were annotated by aligning them with the published plant mitochondrial sequences using BLAST, with manual adjustments made for closely related species; (2) the tRNA genes were annotated using the tRNAscan-SE tool, available at (http://lowelab.ucsc.edu/tRNAscan-SE/, accessed on 18 December 2023) [17]; and (3) the Open Reading Frame Finder (http://www.ncbi.nlm.nih.gov/gorf/gorf.html, accessed on 18 December 2023) was used to identify ORFs, with the minimum length set to 102 base pairs, excluding the redundant sequences and those overlapping with known genes. The sequences with alignments exceeding 300 base pairs were annotated against the NR database. To enhance annotation accuracy, the results were manually reviewed and corrected. Finally, the mtDNA was mapped using OGDRAW (https://chlorobox.mpimp-golm.mpg.de/OGDraw.html, accessed on 18 December 2023).

### 2.4. Codon Usage Analysis

Relative Synonymous Codon Usage (RSCU) is believed to be shaped by a complex interplay of natural selection, mutation pressure, and genetic drift, the numerical value of it is the ratio of the actual frequency of codon usage to the theoretical frequency of codon usage [18]. To refine the dataset and compute the RSCU values, Perl was used to filter the Uniq CDS and perform the calculations.

### 2.5. Analysis of Repeated Sequences

The *N. incerta* mtDNA and cpDNA were found to harbor 3 types of repetitive elements: simple sequence repeats, tandem repeats, and dispersed repeats. To identify these, we employed a variety of tools: (1) Simple sequence repeats were detected using the MIcroSAtellite (MISA, v1.0, parameter:1-102-53-44-35-36-3) identification tool Perl script [19]. (2) Tandem repeats (>6 bp repeat units) were detected using Tandem Repeats Finder v4.09 software (trf409.linux64, parameter: 2778010502000-f-d-m). (3) Dispersed repeats were detected using blastn (v2.10.1). For the visualization of these repeats, Circos version 0.69-5 was employed.

### 2.6. Comparative Analysis of the mtDNA and cpDNA

The homologous gene sequences of *N. incerta* were subjected to global alignment using MAFFT version 7.427 with the “—auto” option. The nucleotide diversity, denoted as Pi, of each gene was then determined using dnasp5 software. For genomic alignment between *N. incerta* and its related species, visualization was performed using nucmer (4.0.0beta2) software. To construct collinearity plots comparing *N. incerta* with its related species, BLASTN version 2.10.1+ was utilized. Moreover, the shared PCGs were aligned using the MAFFT procedure [19]. The Maximum likelihood (ML) phylogenetic tree was conducted by a RAxML v8.2.10 estimation with 1000 bootstrap replications. In addition, to decipher the selective pressures driving the evolution of the genus *N. incerta*, we employed BLASTN to retrieve the homologous protein sequences between *N. incerta* CACC 0356 and its closely related species. Subsequently, the shared PCGs were subjected to multiple sequence alignment using MAFFT version 7 [20]. The non-synonymous (Ka) and synonymous (Ks) ratios (Ka/Ks) were calculated using KaKs Calculator version 2.0 [21].

### 2.7. Chloroplast-to-Mitochondrion DNA Transformation

The homologous gene and tRNA genes, which were transferred from the chloroplasts to the mitochondria, were identified using blast software with the following screening criteria: matching rate 70%, E-value 1 × 10^−5^, and length 30 bp.

## 3. Results

### 3.1. Morphological of N. incerta

**Figure 1:**
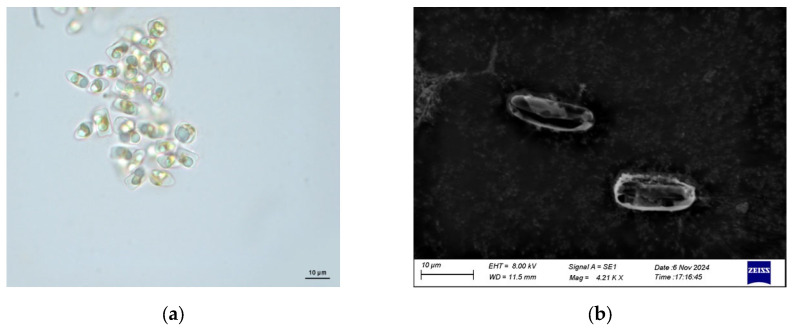
Morphology of diatom *N. incerta* CACC 0356. (**a**) The morphology of *N. incerta* CACC 0356. (**b**) Scanning electron micrograph of *N. incerta* CACC 0356.

The morphology of *N. incerta* CACC 0356 was observed using light and scanning electron microscopy (Figure 1). Cells are typically elongated and boat-shaped, which is characteristic of the genus *Navicula* under light microscopy (Figure 1a). And the cells are small with dimensions in the range of micrometers. Additionally, in SEM, the valves of *N. incerta* are linearlanceolate to lanceolate, with rostrate to subcapitate apices (Figure 1b). The cell wall of *N. incerta* is composed of two valves: an epitheca (upper valve) and a hypotheca (lower valve). The epitheca is generally larger than the hypotheca and overlaps it. *N. incerta* has a girdle composed of the cingulum and the ventral axis, which are the bands that encircle the cell and play a role in cell motility. *N. incerta* contains chlorophyll, which is typically ribbon-shaped or plate-like. The siliceous frustules of *N. incerta* were slightly silicified. The shape of the frustules was elongated or elliptical. The cell size was small (usually less than 8 μm), and it had multiple thin girdle bands, with up to 14 or more. The striae were not visible under the light microscope. The central sternum was prominent and remained in the sample even after the siliceous frustule had been disrupted by standard treatment.

### 3.2. General Features of mtDNA of N. incerta

**Figure 2:**
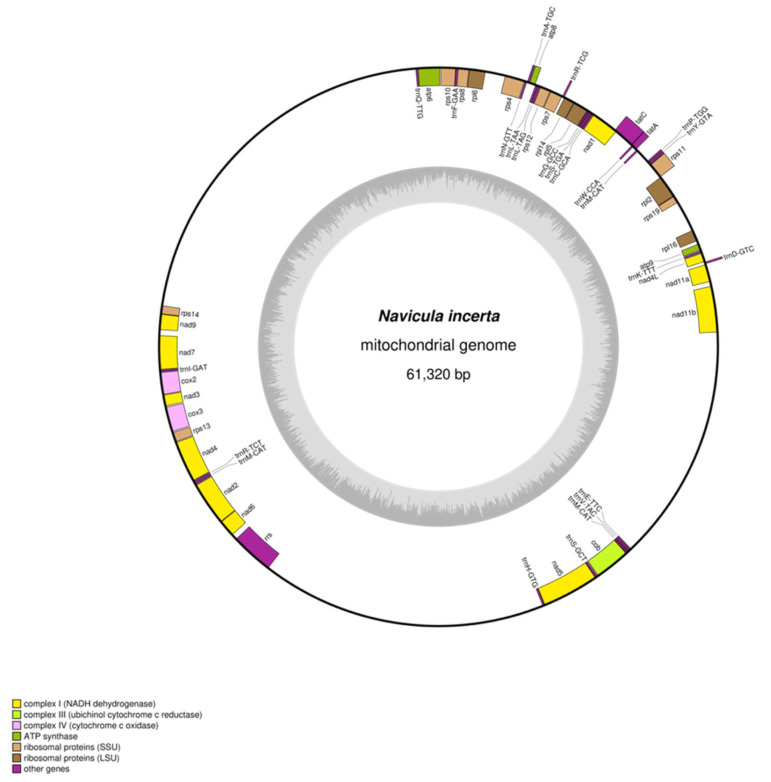
Circular map of the mtDNA of *N. incerta* CACC 0356. Genes transcribed in the forward direction are positioned on the outer perimeter of the circular map, while those transcribed in the opposite direction are situated on the inner side. The inner gray circle depicts the GC content distribution across the mitochondrial genome.

The *N. incerta* library was advanced to next-generation sequencing utilizing an Illumina Novaseq 6000 sequencer, yielding a total of 52,535,221 raw reads. These reads exhibited a GC content of 46.04%, alongside a Q20 score of 97.29% and a Q30 score of 92.77%. Following the elimination of the substandard sequences, a refined set of 1,144,181 high-quality filtered reads was acquired. The reads were subjected to denovo assembly, resulting in a contig consisting of 20,023,053,256 bases. Based on the assembly results, the mtDNA of *N. incerta* was characterized as a single, circular molecular structure spanning a length of 61,320 bp (Figure 2) with a GC content of 29.87%. It contained 60 genes, consisting of 34 mRNA, 24 tRNA, and 2 rRNA genes. Thirty-four PCGs comprised 40.07% of the mtDNA of *N. incerta* CACC 0356. The aggregate length of these genes totaled 24,573 bp. They formed distinct clusters, including those encoding for ATP synthase, ubichinol cytochromec reductase, ctochromec oxidase, NADH dehydrogenase, transport membrance protein, and ribosomal proteins (Table S1). Within the mtDNA of *N. incerta*, the *nad5* and *atp8* genes were the largest and smallest, respectively. The *nad5* gene spanned 2019 bp, constituting 3.29% of the mtDNA, while the *atp8* gene was the most compact at 204 bp, representing a mere 0.33%. All PCGs typically initiated with ATG and terminated with TAA, with the exception of *nad7*, which uniquely started with GTG, and *nad4* and *nad4L*, which concluded with TAG. Meanwhile, the mitochondrial rRNA genes were identified as *rrl* (large subunit, 2807 bp) and *rrs* (small subunit, 1516 bp), collectively contributing a length of 4323 bp, which is 7.05% of the entire mitochondrial genome. The cumulative length of the tRNA genes was 1817 bp, comprising 2.96% of the mtDNA, and no intronic RNA sequences were detected in this assembly.

### 3.3. Anatomization of Repeat Sequence in the mtDNA of N. incerta

**Figure 3:**
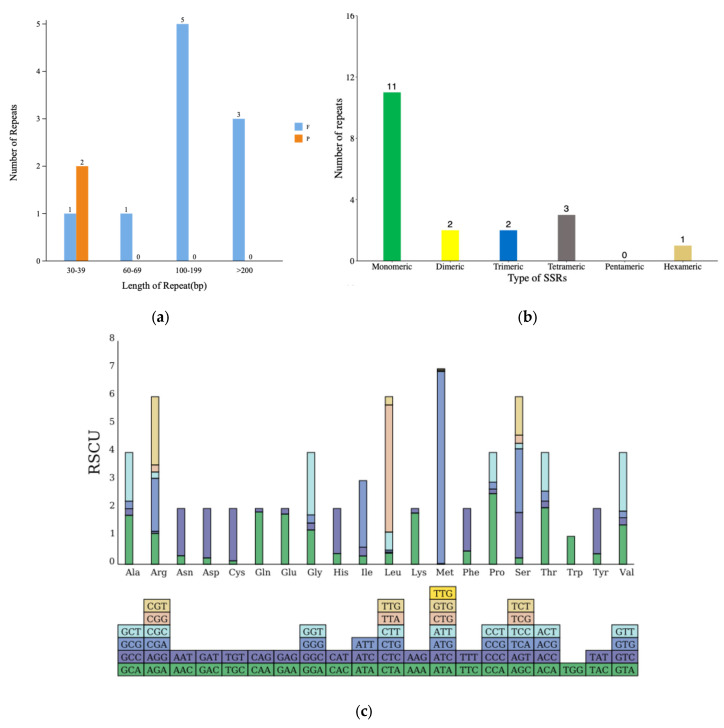

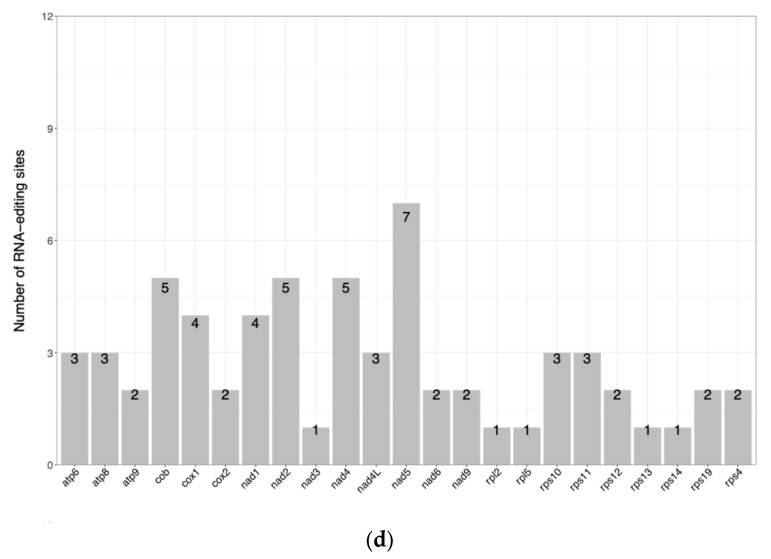
Mitogenome analysis of *N. incerta* CACC 0356. (**a**) Distribution of dispersed repeat lengths. P, palindromic repeat sequences; F, forward repeat sequences. (**b**) Enumeration of identified SSR motifs. (**c**) RSCU analysis. The different amino acids are shown on the x-axis. The x-axis represents different amino acids. The bottom bar corresponds to all the codons encoding each amino acid, with the height of the upper bar indicating the cumulative RSCU values for all the codons. (**d**) Tally of the RNA editing sites identified across each PCG.

During the examination of the repeat sequences within the *N. incerta* mitogenome, our primary focus was directed towards three distinct categories: simple sequence repeats, tandem repeats, and dispersed repeats. A repetitive sequences analysis identified 12 interspersed repeats in the mtDNA of *N. incerta*, including 2 forward and 10 palindromic repeats, ranging from 30 to over 2000 bp in length (Figure 3a). After further splitting, 19 simple sequence repeats (SSRs) were obtained (Figure 3b). Upon further research into the codon usage patterns within the *N. incerta* mtDNA, it was observed that all 71 codons were employed with relative uniformity. Specifically, 29 codons were utilized more extensively (RSCU > 1), while 42 were less frequently selected (RSCU < 1) (Figure 3c and Table S2). Among those with an RSCU value exceeding 1, the initiation codon methionine (ATG) stood out with the highest RSCU of 7.96, indicating its prevalent use. Conversely, the termination codon (TAA) exhibited the most frequent occurrence, with an RSCU of 1.82. Despite a single amino acid being encoded by multiple codons, there existed a notable bias towards certain codons within the mtDNA. For instance, of the four codons encoding glycine, GGA (RSCU = 1.47) and GGT (RSCU = 1.85) were favored more often. Through the analysis and prediction of PCGs, we discovered a total of 64 RNA editing sites across 23 PCGs (Figure 3d). The *atp5* gene harbored the highest number of RNA editing sites, with seven occurrences, while genes such as the *nad3*, *rpl2*, *rpl5*, *rps13*, and *rps14* each contained only a single RNA editing site. The remaining genes featured between two and five editing sites each.

### 3.4. Comparative Analysis of mtDNA of N. incerta and Related Species

**Figure 4:**
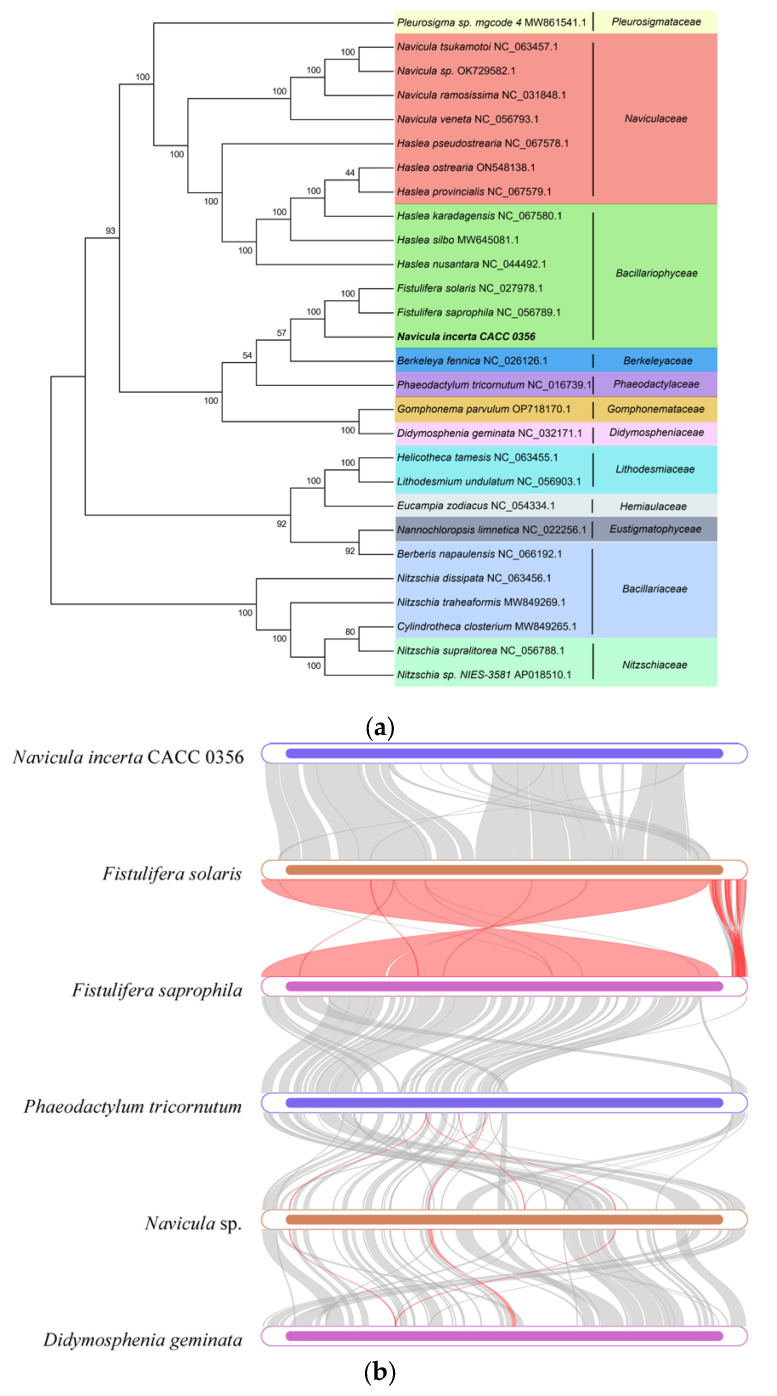

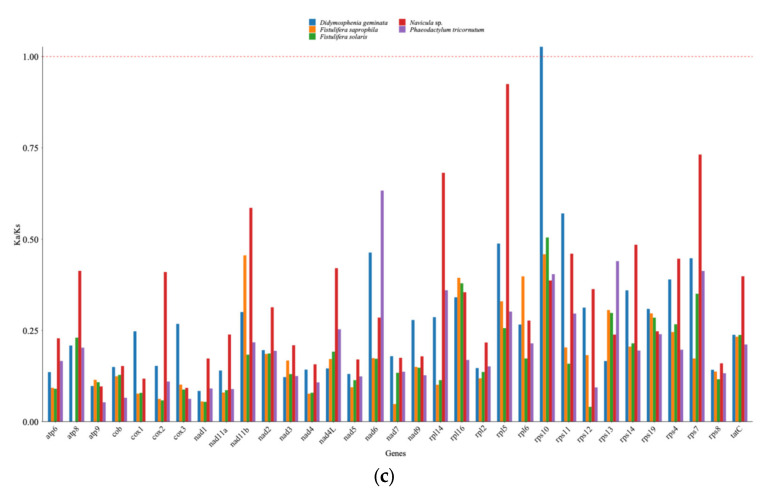
Comparative analysis of mtDNA of *N. incerta* and related species. (**a**) Phylogenetic relationships between *N. incerta* and related species. (**b**) Collinearity plots of the mtDNA of *N. incerta* and related species. The boxes in each row indicate the mitogenomes, and the connecting lines in the middle indicate homologous regions. Red line: inversion alignment; Gray line: anteroposterior alignment. (**c**) Boxplots of the pairwise Ka/Ks values among all *N. incerta* and related species.

To substantiate the taxonomy of the diatoms within the *Navicula* genus using mitogenomic data, we assembled a phylogenetic tree incorporating 27 diatom mitogenomes (Figure 4a), aiming to delve into the evolutionary patterns of mitogenomes across a broad spectrum of species. Based on the information from *Navicula ramosissima*, Naviculales is grouped with Bacillariales. The monophyletic Naviculales included *N. ramosissima*, *Berkeleya fennica*, *Fistulifera solaris*, and *Phaeodactylum tricornutum* [22]. In our study, *N. incerta* CACC 0356 was found to be nestled among the Bacillariaceae species, straddling the boundary between the Naviculaceae and the Berkeleyaceae. The Bacillariaceae species further bifurcated into two distinct sub-branches, one comprising *Fistulifera* sp. and the other encompassing *Haslea* sp. Moreover, numerous homologous collinear blocks were identified between the *N. incerta* mitochondrial genome and its five closest relatives (Figure 4b). The gaps within these blocks suggest the presence of species-specific sequences that lack homology with the other species, indicating substantial genomic rearrangements within the mitochondrial genomes of closely related species. There were high similarities in the mitochondrial structures of *N. incerta* and *Fistulifera solaris* (NCBI Number: 027978.1), *Fistulifera saprophila* (NCBI Number: 056789.1), *Phaeodaetylum trieornutum* (NCBI Number: 016739.1), and *Didosphenia geminata* (NCBI Number: 032171.1). This analysis underscores the utility of mitogenomes in elucidating the phylogenetic relationships and genetic affinities among species. Furthermore, the Ka/Ks ratio, which compares the rate of Ka to Ks, serves as a metric for gauging the selective pressures on proteins during evolution [23]. A Ka/Ks ratio greater than one signifies positive selection, a ratio of one indicates neutral evolution, and a ratio of less than one suggests negative or purifying selection [24].To assess the selective pressures acting on the PCGs in *N. incerta* and its closely related species, we determined the Ka/Ks values for 33 mitochondrial genes. The findings, depicted in Figure 4c, reveal that 30 mitochondrial PCGs exhibited Ka/Ks values of less than one, suggesting that these genes have undergone purifying selection, thereby maintaining stable protein functions. Conversely, the average Ka/Ks value for *rps10* exceeded one (Ka/Ks = 1.02671), which was strongly and positively selected.

**Figure 5:**
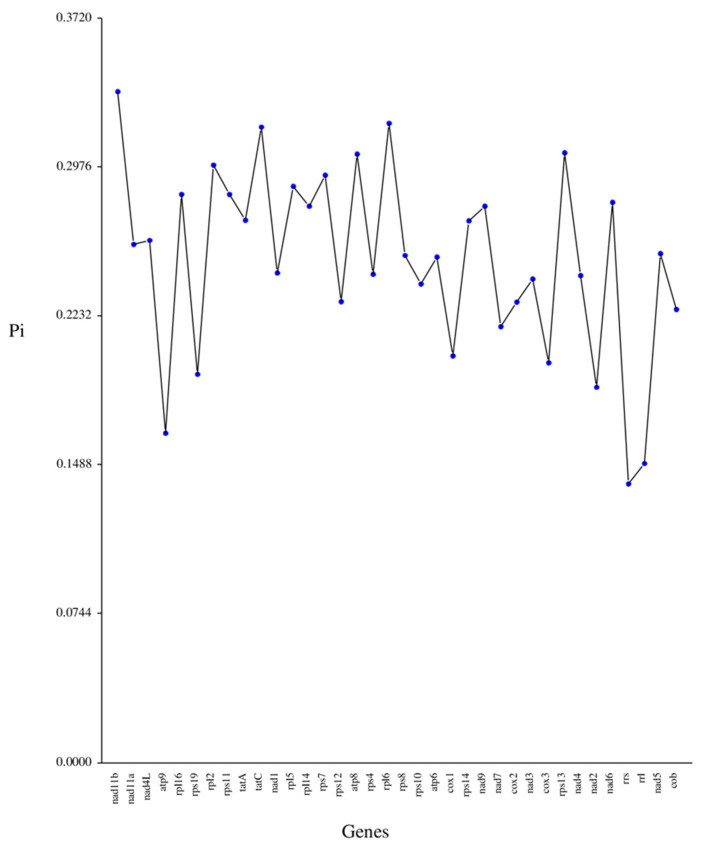
Nucleotide diversity (Pi) among 36 protein-coding genes in mtDNA of *N. incerta*.

Concurrently, Pi serves as a valuable metric for assessing genetic disparities in nucleotide sequences across various species and populations. Consequently, the regions exhibiting heightened variability can be identified and employed as potential molecular markers for population studies [25]. The Pi values across the mitochondrial genes varied from 0.13898 to 0.33522, with no genes having a value below 0.10 (Figure 5 and Table S3). The gene *nad11b*, with a Pi value of 0.33352, showed the highest degree of variability, closely followed by *rpl6* (Pi = 0.31935) and *tatC* (Pi = 0.31746), as well as *rps13* (Pi = 0.3046), all of which demonstrated significant variability. This suggests that the nucleotide sequences of the majority of the mitochondrial genes in *N. incerta* are not highly conserved.

### 3.5. General Features of cpDNA of N. incerta

**Figure 6:**
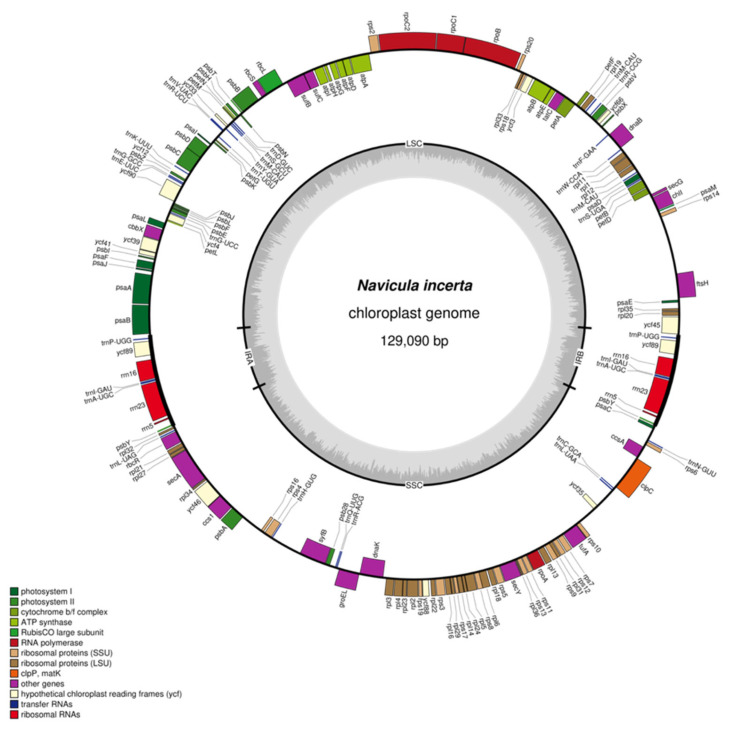
Circular map of the cpDNA of *N. incerta* CACC 0356.

We assembled the complete circular cpDNA of *N. incerta* CACC 0356, which was a 129,090 bp circular chromosome (Figure 6). The cpDNA of *N. incerta* displays a classic quadripartite structure, comprising a pair of inverted repeat (IR) regions, designated as IRa and IRb, each spanning 7331 base pairs. These IR regions are flanked by a large single-copy (LSC) region of 67,707 base pairs and a small single-copy (SSC) region of 46,721 base pairs. The overall GC content of the cpDNA is 30.81%, with distinct variations across the different regions: IRa/IRb at 40.94%, LSC at 29.47%, and SSC at 29.56% (as detailed in Table S4). The annotation of the cpDNA disclosed the presence of 127 protein-coding genes (PCGs), 30 tRNA genes, and 6 rRNA genes, all of which were intron-less (Table 1). These genes are categorized into several functional groups, including those involved in photosynthesis, self-replication, other essential functions, and a subset with unknown functions.

### 3.6. Anatomization of Repeat Sequence in the cpDNA of N. incerta

**Figure 7:**
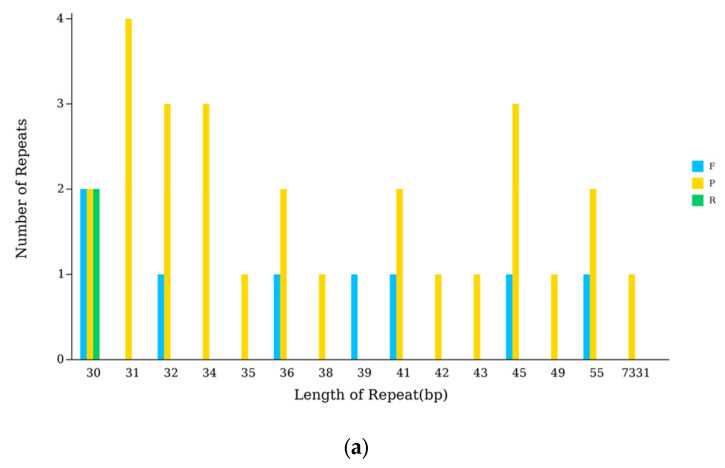

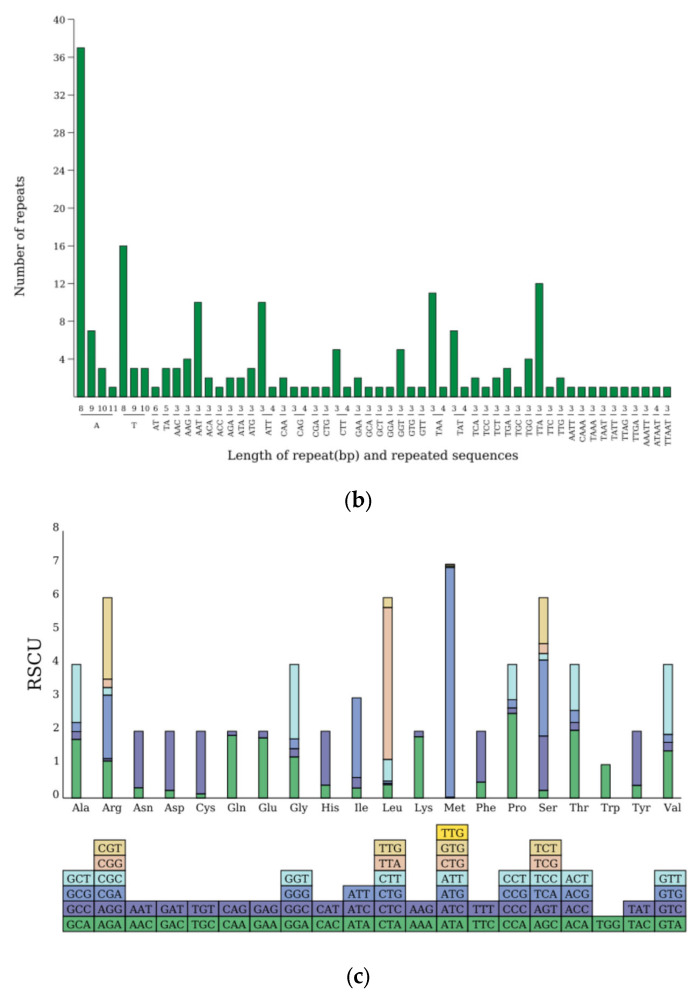
The cpDNA analysis of *N. incerta* CACC 0356. (**a**) Numbers of repetitive sequences. P, palindromic repetition; F, forward repetition; R, reverse cleotide; C, complementary repetition. (**b**) Numbers and types of SSR. (**c**) RSCU analysis.

An analysis with the Reputer tool uncovered 37 dispersed repeats within the cpDNA of *N. incerta*, consisting of 8 forward, 27 palindromic, 2 reverse, and no complementary repeats. These repeats spanned a length spectrum from 30 to 7331 bp (Figure 7a). Additionally, 194 SSRs were identified, which included 70 mononucleotide, 4 dinucleotide, 110 trinucleotide, 7 tetranucleotide, and 5 pentanucleotide repeats (Figure 7b). The trinucleotide repeats, with a predominance of A or T, were the most frequently observed. Furthermore, the codon usage analysis indicated that Leu was the most frequently occurring amino acid, with 3,205 instances, followed by Ile with 2,661 occurrences, while Ter was the least common, appearing only 127 times (Figure 7c). The RSCU values peaked for the Leu codon UUA at 4.5492 and reached their nadir for the Leu codon CUC at 0.00396. Among the 29 codons exhibiting RSCU values greater than one, the majority concluded with A or U, reflecting a preference. The codon for Tryptophan (UGG) was the sole exception, showing no bias with an RSCU value of one (Table S5). By aligning the transcriptome data with the cpDNA, 64 RNA editing sites were pinpointed within the chloroplast genes of *N. incerta* CACC 0356 (Table 2).

### 3.7. Comparative Analysis of cpDNA of N. incerta and Related Species

**Figure 8:**
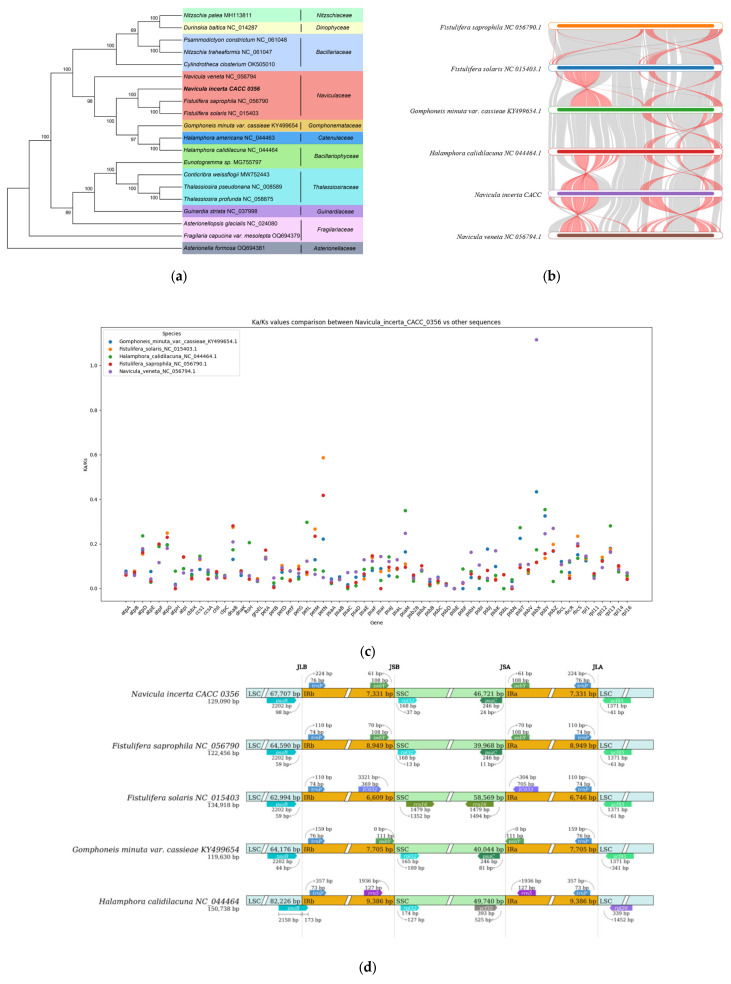
Comparative analysis of cpDNA of *N. incerta* and related species. (**a**) Phylogenetic relationships between *N. incerta* and related species. (**b**) Collinearity plots of the cpDNA of *N. incerta* and related species. Red line: inversion alignment; Gray line: anteroposterior alignment. (**c**) Boxplots of the pairwise Ka/Ks values among all *N. incerta* and related species. (**d**) Analysis of chloroplast IR boundary changes.

The ML phylogenetic tree of the *N. incerta* was conducted based on the 19 concatenated proteins derived from all known cpDNAs, as illustrated in Figure 8a. The analysis yielded robust support, with the majority of nodes receiving a 100% confidence score, and the remaining nodes garnering support of at least 69%. The results revealed that *N. incerta* CACC 0356 is closely related to *Fistulifera saprophila* and *Fistulifera solaris*, which belong to the Naviculaceae order. Phylogenetic analyses based on the genomic data from both the chloroplast and mitochondrial genomes have consistently indicated that *N. incerta* shares a closer affinity with *Fistulifera saprophila* than with any other known diatom species. Additionally, there were six homologous collinear blocks between the *N. incerta* mtDNA and the other five related species (Figure 8b). Furthermore, the average Ka/Ks value for the chloroplast gene rpl34 exceeded one, suggesting positive selection, while the Ka/Ks values for the remaining 619 genes were below one. This indicates that the majority of the PCGs in the chloroplasts were subject to negative selection and have been highly conserved throughout evolution. These insights underscore the varying evolutionary pressures acting on specific genes within the Navicula chloroplasts, with the majority being conserved and a select few potentially undergoing adaptive evolution [26]. Also, the IR/LSC and IR/SSC boundary regions of four Navicula species were examined (Figure 8d). The lengths of the cpDNA spanned from 119,630 bp (*Gomphoneis minuta varcassieae*) to 150,738 bp (*Halamphora calidilacuna*), with all species exhibiting similar tetrameric region boundary structures. Especially, the length from the *psaB* gene to the JLB region of *N. incerta* which was induced to 98 bp, while this distance ranged from 44 bp to 59 bp in the other three species (*Fistulifera saprophila* and *Fistulifera solaris*, *Gomphoneis minuta varcassieae*). This variation suggests distinct evolutionary trajectories and potential functional divergences within the chloroplast genomes of these species. Specifically, the length from the rrn16 gene to the JSB boundary was only found in *Fistulfera solaris*, and the other four species had *rpl32* genes with 13 bp to 127 bp. In the context of the JLA boundary, the distances from the *trnP* genes across all five species were found to vary, falling within a narrow range of 73 to 76 bp. Despite these minor fluctuations, the overall architecture of the IR boundaries showed a high degree of conservation, indicating a stable and preserved structural framework among these species.

**Figure 9:**
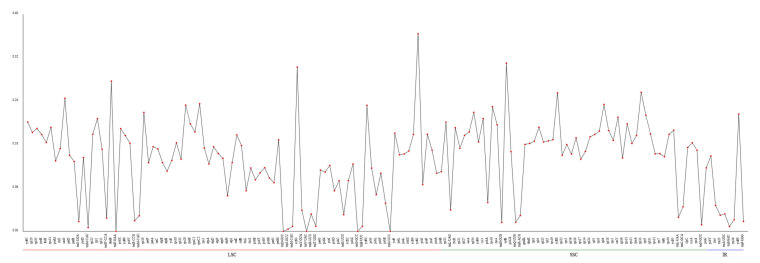
Nucleotide diversity (Pi) in the chloroplast protein-coding genes of *N. incerta*.

The Pi values of the chloroplast genes ranged from 0 to 0.36394 in the LSC region, 0.01204 to 0.30985 in the SSC region, and 0.00901 to 0.216 in the IR region (Figure 9 and Table S6). The Pi analysis across the cpDNA of *N. incerta* indicated significant variability in the LSC and SSC regions, particularly in *ycf41* (Pi = 0.36394) and *syfB* (Pi = 0.30985), respectively. Also, the positive selection of the *rpl34* gene indicated moderate variability (Pi = 0.19142). There were 41 genes within the Pi < 0.1 range in the cpDNA of *N. incerta*, which was more than that of the mtDNA. Consequently, the Pi values suggest that the nucleotide sequence of the chloroplast gene in *N. incerta* exhibits greater conservation when compared to its mitochondrial counterpart.

### 3.8. Sequence Similarity Between the mtDNA and cpDNA of N. incerta

**Figure 10:**
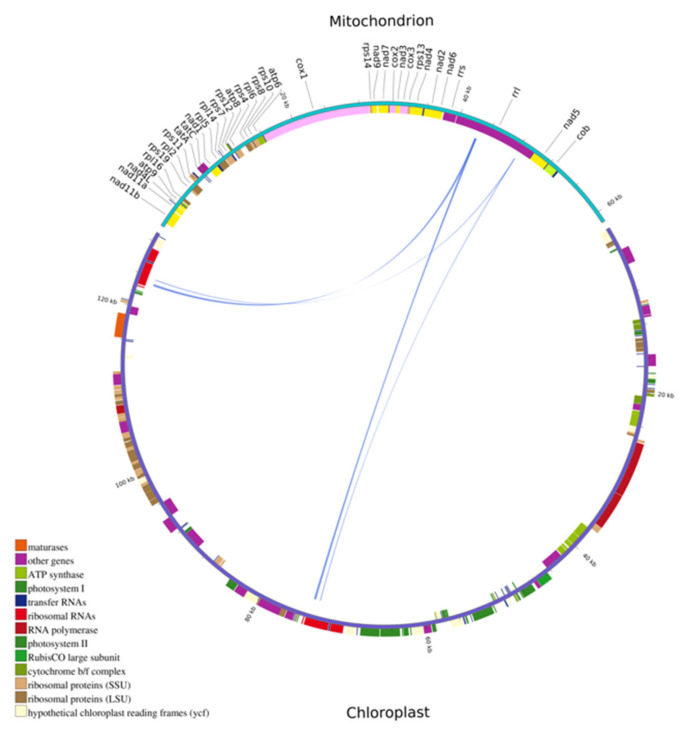
Distribution of homologous fragments between mtDNA and cpDNA of *N. incerta*. Genes from the same complex are color-coded, with homologous sequences indicated at the center line junctions.

The cpDNA of *N. incerta* was approximately 2.1 times longer than its mtDNA counterpart. After annotating these homologous sequences, four genes were found to be partially located in the homologous sequences between the mitogenome and chloroplast genome with a total length of 372 bp, accounting for 0.61% of the mtDNA (Figure 10 and Table S7). According to the amino acid sequence similarity analysis, there were 13 transferred genes in *N. incerta* (Table S8). The length of the *rpl2*_len275 gene was the longest with a length of 189 bp. The *rps19*_len94 gene had the shortest length (79 bp).

## 4. Discussion

Though diatoms are believed to encompass an estimated 200,000 species, only approximately 10,000 have been documented and annotated to date [27]. This means that a majority of the diatom species are yet to be discovered and classified. The genomes of organelles such as mitochondria and chloroplast exhibit a significant degree of variation, and have independent heritability for distinguishing different marine diatom species [28]. Currently, our understanding of the structure and function of diatom organelle genomes remains limited, which significantly impedes further research and the practical application of diatoms. In this study, a single circular molecule of 61,320 bp in length for the *N. incerta* mtDNA was used to complement and fill the gaps in the current understanding the genetic makeup of *N. incerta*. Meanwhile, the cpDNA of *N. incerta* also revealed a single circular molecule spanning 129,090 bp and containing 163 genes. Our findings on *N. incerta* differ from the previous plant studies, where the cpDNA of plants was smaller in size, more structurally stable, and exhibited higher sequence conservation when compared with the mtDNA [29]. However, the length of the cpDNA was approximately 2.1 times longer than that of the mtDNA of *N. incerta* CACC 0356, which showed frequent DNA fragment flow between the mitochondria and chloroplasts. Although mtDNA and cpDNA are relatively conserved, there are also more differences in the mtDNA and cpDNA of different species.

According to the mtDNA of *N. incerta*, we discovered a total of 35 repetitive sequences, which sum up to a length of 2796 base pairs, accounting for over 4.5% of the mtDNA. These repetitive sequences lay the groundwork for molecular recombination events in the mitochondria, which can involve varying sizes and quantities of DNA segments [30,31]. Similarly, the repetitive sequences were no more than 10% in *Navicula ramosissima* TA439 (48,652 bp) [22] and in *Navicula vanseea* (43,997 bp) of the entire mitogenome [32]. This suggests that the mtDNA of *N. incerta* possesses a lower frequency of repetitive sequences and sequence transpositions, potentially indicating a more conserved state throughout the evolutionary history of diatoms. Additionally, we observed that the prevalence of codons with a preference for A/T at the third base position was significantly higher than those ending with G/C, a pattern commonly seen in most species [33]. This points to a distinct preference for base changes in *N. incerta*’s mtDNA genes. Furthermore, while the number of core genes is relatively modest (34 PCGs), there is a high prevalence of variable genes. These variations are attributed to the loss and transference of the PCGs within the mitogenome throughout the course of evolution. the genomes of plastids and mitochondria often experience DNA fragment transfer throughout evolution [22,34]. Also, we found 64 RNA editing sites in the mtDNA. RNA editing can also induce specific and significant alterations in the expression patterns of mtDNA genes [35]. Consequently, by examining the number of mitochondrial PCGs annotated in *N. incerta*, we inferred that substantial genetic shifts occurred in the evolutionary trajectory of the diatoms from a common ancestor, likely to enhance their adaptability to their environment.

Diatoms are categorized into two major subdivisions: the Coscinodiscophytina, and the Bacillariophytina, comprising the Mediophyceae group and the Bacillariophyceae group, known for pennate diatoms [36,37]. A phylogenetic analysis of whole mtDNA from 27 related species positioned *N. incerta* within Bacillariaceae, closely related to *Fistulifera* sp. Additionally, the Ka/Ks ratio showed a potential positive selection in the *rps10* gene. And the *rps10* gene (Pi = 0.23893) exhibited great variability. The *rps10* gene encoded the S10 protein which is part of the small subunit of mitochondrial ribosomes [38]. Over 90% of the genes exhibited Ka/Ks values below one, indicating that the genes associated with ATP synthase, cytochrome c oxidase, and NADH dehydrogenase were predominantly under the influence of purifying selection, maintaining a conservative state throughout the evolutionary process in comparison to *N. incerta*.

Significantly, certain cpDNA genes, including *rpl32*, *rpl20*, and *psbY*, were found to be absent in the common ancestor of Bacillariophyceae. Prior research has indicated that the loss of chloroplast genes is a frequent occurrence in the evolution of diatoms [39]. Interestingly, the multigene phylogenetic analysis, leveraging both mtDNA and cpDNA, affirmed the distant evolutionary relationship between *N. incerta* and the *Nitzschiaceae* family. Furthermore, the Ka/Ks ratio analysis suggested the *rpl34* gene in chloroplasts was a positive selection, which indicated that the gene may have undergone the action of natural selection during evolution [40]. The *rpl34* gene, which encodes a cytoplasmic ribosomal protein highly homologous to the rat 60S r-protein L34, was extracted from a genomic library of tobacco [41]. Moreover, the variation in the size of cpDNA is partly attributed to the contraction and expansion of the IR region, leading to a significant number of gene duplications in the diatoms [42]. The IR regions across five diatoms’ plastid genomes, ranging from 7 to 9 kb, suggest a relatively conserved nature within the Bacillariaceae group.

In the selection pressure analysis across species, different trends were obtained based on the mtDNAs and cpDNAs [43]. When contrasted with *Fistulfera saprcphila*, *N. incerta* had the lowest Ka/Ks ratio in the *rps10* gene, suggesting that the *rps10* gene had evolved under comparable selection pressures to *Fistulfera saprcphila*. On the other hand, compared to *Navicula veneta* cpDNA, the Ka/Ks ratios of the *rpl34* and *psbX* genes in *N. incerta* were considerably higher. This indicates that *N. incerta* was subjected to more selection pressure, resulting in adaptive changes within the sequences of the *rpl34* and *psbX* genes.

## 5. Conclusions

This study presents the first sequencing, assembly, and annotation of the mtDNA and cpDNA of *N. incerta* CACC 0356. The total length of the mtDNA was 61320 bp, with a GC content of 29.87%. It consists of one circular DNA, including 34 protein-coding genes, 24 tRNAs, and 34 rRNAs, while the circular cpDNA was 129090 bp, containing 127 PCGs, 30 tRNAs, and 7 rRNAs. The cpDNA of *N. incerta* was approximately 2.1 times longer than its mtDNA counterpart. After annotating these homologous sequences, there were four genes partially located in the homologous sequences between the mtDNA and cpDNA with a total length of 372 bp, accounting for 0.61% of the mtDNA. This research not only deepens our understanding of species diversity but also offers novel perspectives on the evolutionary history of the Bacillariophyceae species. Future studies may focus on the detailed analysis of its genome to further understand its biological properties and improve its efficiency in various applications.

## Figures and Tables

**Table 1:** Functional classification of the genes of the *N. incerta* CACC 0356 chloroplast genome.

Category	Gene Group	Gene Name
Photosynthesis	Subunits of photosystem I	*psaA*, *psaB*, *psaC*, *psaD*, *psaE*, *psaF*, *psaI*, *psaJ*, *psaL*, *psaM*
	Subunits of photosystem II	*psb28*, *psbA*, *psbB*, *psbC*, *psbD*, *psbE*, *psbF*, *psbH*, *psbI*, *psbJ*, *psbK*, *psbL*, *psbN*, *psbT*, *psbV*, *psbX*, *psbY(2)*, *psbZ*
	Subunits of NADH dehydrogenase	*-*
	Subunits of cytochrome b/f complex	*petA*, *petB*, *petD*, *petF*, *petG*, *petL*, *petM*, *petN*
	Subunits of ATP synthase	*atpA*, *atpB*, *atpD*, *atpE*, *atpF*, *atpG*, *atpH*, *atpI*
	Large subunit of rubisco	*rbcL*, *rbcR*, *rbcS*
	Subunits of photochlorophyllide reductase	*chlI*
Self-replication	Proteins of large ribosomal subunit	*rpl1*, *rpl11*, *rpl12*, *rpl13*, *rpl14*, *rpl16*, *rpl18*, *rpl19*, *rpl2*, *rpl20*, *rpl21*, *rpl22*, *rpl23*, *rpl24*, *rpl27*, *rpl29*, *rpl3*, *rpl31*, *rpl32*, *rpl33*, *rpl34*, *rpl35*, *rpl36*, *rpl4*, *rpl5*, *rpl6*
	Proteins of small ribosomal subunit	*rps10*, *rps11*, *rps12*, *rps13*, *rps14*, *rps16*, *rps17*, *rps18*, *rps19*, *rps2*, *rps20*, *rps3*, *rps4*, *rps5*, *rps6*, *rps7*, *rps8*, *rps9*
	Subunits of RNA polymerase	*rpoA*, *rpoB*, *rpoC1*, *rpoC2*
	Ribosomal RNAs	*rrn16(2)*, *rrn23(2)*, *rrn5(2)*
	Transfer RNAs	*trnA-UGC(2)*, *trnC-GCA*, *trnD-GUC*, *trnE-UUC*, *trnF-GAA*, *trnG-GCC*, *trnG-UCC*, *trnH-GUG*, *trnI-GAU(2)*, *trnK-UUU*, *trnL-UAA*, *trnL-UAG*, *trnM-CAU(3)*, *trnN-GUU*, *trnP-UGG(2)*, *trnQ-UUG*, *trnR-ACG*, *trnR-CCG*, *trnR-UCU*, *trnS-GCU*, *trnS-UGA*, *trnT-UGU*, *trnV-UAC*, *trnW-CCA*, *trnY-GUA*
Other genes	Maturase	*-*
	Protease	*clpC*
	Envelope membrane protein	*-*
	Acetyl-CoA carboxylase	*-*
	c-type cytochrome synthesis gene	*ccs1*, *ccsA*
	Translation initiation factor	*-*
	other	*cbbX*, *dnaB*, *dnaK*, *ftsH*, *groEL*, *secA*, *secG*, *secY*, *sufB*, *sufC*, *syfB*, *tatC*, *tufA*
Genes of unknown function	Conserved hypothetical chloroplast ORF	*ycf12*, *ycf3*, *ycf33*, *ycf35*, *ycf39*, *ycf4*, *ycf41*, *ycf45*, *ycf46*, *ycf66*, *ycf88*, *ycf89(2)*, *ycf90*

**Table 2:** Prediction of RNA editing sites.

Type	RNA-Editing	Number	Percentage
hydrophilic–hydrophilic	CAT (H) ⇒ TAT (Y)	2	
	total	2	3.12%
hydrophilic–hydrophobic	ACA (T) ⇒ ATA (I)	4	
	ACC (T) ⇒ ATC (I)	1	
	ACT (T) ⇒ ATT (I)	14	
	TCA (S) ⇒ TTA (L)	4	
	TCG (S) ⇒ TTG (L)	1	
	TCT (S) ⇒ TTT (F)	2	
	total	26	40.62%
hydrophobic–hydrophilic	CCA (P) ⇒ TCA (S)	4	
	CCC (P) ⇒ TCC (S)	1	
	CCT (P) ⇒ TCT (S)	3	
	total	8	12.50%
hydrophobic–hydrophobic	CCA (P) ⇒ CTA (L)	1	
	CCC (P) ⇒ CTC (L)	1	
	CCT (P) ⇒ TTT (F)	4	
	CTC (L) ⇒ TTC (F)	1	
	CTT (L) ⇒ TTT (F)	4	
	GCA (A) ⇒ GTA (V)	5	
	GCC (A) ⇒ GTC (V)	1	
	GCG (A) ⇒ GTG (V)	1	
	GCT (A) ⇒ GTT (V)	10	
	total	28	43.75%
	All	64	100%

## Data Availability

The data generated in this article are available in the GenBank databases (https://www.ncbi.nlm.nih.gov/, accessed 28 November 2024) with accession numbers PQ722531 and PQ722532.

## Supplementary Materials

The following supporting information can be downloaded at https://www.mdpi.com/article/10.3390/life15010102/s1: Table S1: Functional classification of genes and physical location of the *N. incerta* CACC 0356 mitogenome; Table S2: RSCU values and numbers for codons in the CDS analysis of the mtDNA in *N. incerta*; Table S3: The nucleotide variability of *N. incert* mitogenome; Table S4: The characteristics of *N. incerta* chloroplast genome; Table S5: RSCU values and numbers for codons in the CDS analysis of the *N. incerta* chloroplast genome; Table S6: The nucleotide variability of *N. incert* chloroplast genome; Table S7: Comparison information of the mtDNA and cpDNA in *N. incerta*; and Table S8: Transferred genes between the mtDNA and cpDNA of *N. incerta*.
