# Supplementary material for: Complete Sequence and Characterization of Mitochondrial and Chloroplast Genome of Navicula incerta CACC 0356
Source: Life (Basel). 2025 Jan 15;15(1):102. doi: 10.3390/life15010102 (PMC11767216; doi:10.3390/life15010102)
Supplement: Supplementary file 1 [file life-15-00102-s001.zip › life-3382270-supplementary.pdf]

Supplementary information

Complete Sequence and Characterization of Mitochondrial and Chloroplast Genome of *Navicula incerta* CACC 0356

Zhaokai Wang and Xiaoyu Wei *

Technical Innovation Center for Utilization of Marine Biological Resources, Third Institute of Oceanography, Ministry of Natural Resources, Xiamen 361000, China
* Correspondence: weixiaoyu@tio.org.cn

Table S1. Functional classification of genes and physical location of the *N. incerta* CACC 0356 mitogenome.

Group of genes		Gene name	Length	Location		No. of Amino acid
				Start codon	Stop codon	
mRNA	ATP synthase	atp6	768	ATG	TAA	256
		atp8	204	ATG	TAA	68
		atp9	228	ATG	TAA	76
	Ubichinol cytochrome c reductase	cob	1263	ATG	TAA	421
	Cytochromec oxidase	cox1	1473	ATG	TAA	491
		cox2	786	ATG	TAA	262
		cox3	888	ATG	TAA	296
	Transport membrance protein	tatA	231	ATG	TAA	77
		tatC	780	ATG	TAA	260
	NADH dehydrogenase	nad1	987	ATG	TAA	329
		nad11a	624	ATG	TAA	208
		nad11b	1629	ATG	TAA	543
		nad2	1548	ATG	TAA	516
		nad3	402	ATG	TAA	134
		nad4	1470	ATG	TAG	490
		nad4L	321	ATG	TAG	107
		nad5	2019	ATG	TAA	673
		nad6	603	ATG	TAA	201
		nad7	1197	GTG	TAA	399
		nad9	558	ATG	TAA	186
	LSU Ribosomal proteins	rpl14	381	ATG	TAA	127
		rpl16	405	ATG	TAA	135
		rpl2	810	ATG	TAA	270
		rpl5	537	ATG	TAA	179
		rpl6	567	ATG	TAA	189
	SSU Ribosomal proteins	rps10	528	ATG	TAG	176
		rps11	546	ATG	TAA	182
		rps12	381	ATG	TAA	127
		rps13	348	ATG	TAA	116
		rps14	300	ATG	TAA	100
		rps19	261	ATG	TAA	87
		rps4	708	ATG	TAA	236
		rps7	435	ATG	TAA	145
		rps8	387	ATG	TAA	129

rRNA	Ribosomal RNAs	rrl	2807
		rrs	1516
tRNA	Transfer RNAs	trnA-TGC	73
		trnC-GCA	73
		trnD-GTC	74
		trnE-TTC	72
		trnF-GAA	73
		trnG-GCC	73
		trnH-GTG	74
		trnI-GAT	74
		trnK-TTT	74
		trnL-TAA	84
		trnL-TAG	85
		trnM-CAT	72
		trnM-CAT	74
		trnM-CAT	73
		trnN-GTT	72
		trnP-TGG	72
		trnQ-TTG	72
		trnR-TCG	74
		trnR-TCT	74
		trnS-GCT	90
		trnS-TGA	86
		trnV-TAC	73
		trnW-CCA	73
		trnY-GTA	83

Table S2. RSCU values and numbers for codons in the CDS analysis of the mtDNA in *N. incerta*.

AminoAcid	Codon	No.	RSCU	AminoAcid	Codon	No.	RSCU
Ter	UAA	31	1.8236	Met	AUU	0	0
Ter	UAG	3	0.1764	Met	CUG	0	0
Ala	GCA	126	1.3404	Met	GUG	1	0.044
Ala	GCC	26	0.2764	Met	UUA	0	0
Ala	GCG	27	0.2872	Met	UUG	0	0
Ala	GCU	197	2.0956	Asn	AAC	105	0.4242
Cys	UGC	16	0.3076	Asn	AAU	390	1.5758
Cys	UGU	88	1.6924	Pro	CCA	88	1.3592
Asp	GAC	36	0.3396	Pro	CCC	15	0.2316
Asp	GAU	176	1.6604	Pro	CCG	15	0.2316
Glu	GAA	224	1.697	Pro	CCU	141	2.1776
Glu	GAG	40	0.303	Gln	CAA	169	1.779
Phe	UUC	104	0.2896	Gln	CAG	21	0.221
Phe	UUU	614	1.7104	Arg	AGA	147	3.4452
Gly	GGA	163	1.4684	Arg	AGG	17	0.3984
Gly	GGC	22	0.198	Arg	CGA	47	1.1016
Gly	GGG	54	0.4864	Arg	CGC	2	0.0468
Gly	GGU	205	1.8468	Arg	CGG	10	0.2346
His	CAC	37	0.4836	Arg	CGU	33	0.7734
His	CAU	116	1.5164	Ser	AGC	22	0.2154
Ile	AUA	243	0.9264	Ser	AGU	169	1.6566
Ile	AUC	63	0.2403	Ser	UCA	172	1.686
Ile	AUU	481	1.8336	Ser	UCC	15	0.147
Lys	AAA	609	1.7992	Ser	UCG	42	0.4116
Lys	AAG	68	0.2008	Ser	UCU	192	1.8822
Leu	CUA	81	0.462	Thr	ACA	155	1.4156
Leu	CUC	5	0.0288	Thr	ACC	28	0.2556
Leu	CUG	13	0.0744	Thr	ACG	21	0.1916
Leu	CUU	133	0.7584	Thr	ACU	234	2.1368
Leu	UUA	705	4.0212	Val	GUA	170	1.4624
Leu	UUG	115	0.6558	Val	GUC	18	0.1548
Met	AUA	0	0	Val	GUG	28	0.2408
Met	AUC	0	0	Val	GUU	249	2.142
Met	AUG	182	7.956	Trp	UGA	1	0.0174
Met	AUU	0	0	Trp	UGG	114	1.9826
Met	CUG	0	0	Tyr	UAC	82	0.4594
Met	GUG	1	0.044	Tyr	UAU	275	1.5406
Met	AUG	182	7.956				

Table S3. The nucleotide variability of *N. incert* mitogenome.

Number	Region	Pi	Total Number of mutations	Region length
1	gene1.nad11b	0.33522	571	1636
2	gene10.tatC	0.31746	359	798
3	gene11.nad1	0.24451	464	987
4	gene12.rpl5	0.28782	253	549
5	gene13.rpl14	0.27787	205	402
6	gene14.rps7	0.29341	213	463
7	gene15.rps12	0.2301	174	387
8	gene16.atp8	0.30392	88	207
9	gene17.rps4	0.24388	255	720
10	gene18.rpl6	0.31935	317	579
11	gene19.rps8	0.25326	190	402
12	gene2.nad11a	0.25883	278	1149
13	gene20.rps10	0.23893	181	535
14	gene21.atp6	0.25249	379	780
15	gene22.cox1	0.20297	551	1509
16	gene23.rps14	0.27056	134	342
17	gene24.nad9	0.27786	292	861
18	gene25.nad7	0.21766	508	1197
19	gene26.cox2	0.22992	307	813
20	gene27.nad3	0.24145	186	444
21	gene28.cox3	0.19951	326	891
22	gene29.rps13	0.3046	190	351
23	gene3.nad4L	0.2608	169	321
24	gene30.nad4	0.24317	705	1482
25	gene31.nad2	0.18734	420	1731
26	gene32.nad6	0.27986	300	621
27	gene33.rrs	0.13898	429	2006
28	gene34.rrl	0.14921	839	3019
29	gene35.nad5	0.2542	987	2031
30	gene36.cob	0.22627	413	1314
31	gene4.atp9	0.16433	77	234
32	gene5.rpl16	0.28379	212	408
33	gene6.rps19	0.1938	74	261
34	gene7.rpl2	0.2984	405	817
35	gene8.rps11	0.28374	253	555
36	gene9.tatA	0.27083	39	231

Table S4. The characteristics of *N. incerta* chloroplast genome.

Category	Item	Describe
Chloroplast genome structure	Total length (bp)	129090
	LSC length (bp)	67707
	SSC length (bp)	46721
	IRa/IRb length (bp)	7331
Gene composition	Gene number	163
	tRNA	30
	rRNA	6
	Protein-coding genes	127
GC content	Chloroplast gene	30.81%
	LSC	29.47%
	SSC	29.56%
	IRa/IRb	40.94%

Table S5. RSCU values and numbers for codons in the CDS analysis of the *N. incerta* chloroplast genome.

Amino Acid	Codon	No.	RSCU	Amino Acid	Codon	No.	RSCU
Ter	UAA	116	2.7402	Met	AUU	3	0.0371
Ter	UAG	10	0.2361	Met	CUG	0	0
Ter	UGA	1	0.0237	Met	GUG	4	0.049
Ala	GCA	805	1.7576	Met	UUG	1	0.0126
Ala	GCC	107	0.2336	Asn	AAC	261	0.3062
Ala	GCG	125	0.2728	Asn	AAU	1444	1.6938
Ala	GCU	795	1.736	Pro	CCA	720	2.5352
Cys	UGC	15	0.1234	Pro	CCC	46	0.162
Cys	UGU	228	1.8766	Pro	CCG	70	0.2464
Asp	GAC	139	0.228	Pro	CCU	300	1.0564
Asp	GAU	1080	1.772	Gln	CAA	1077	1.8746
Glu	GAA	1587	1.8004	Gln	CAG	72	0.1254
Glu	GAG	176	0.1996	Arg	AGA	263	1.1088
Phe	UUC	336	0.4752	Arg	AGG	17	0.0714
Phe	UUU	1078	1.5248	Arg	CGA	451	1.9014
Gly	GGA	594	1.2312	Arg	CGC	54	0.2274
Gly	GGC	119	0.2468	Arg	CGG	60	0.2532
Gly	GGG	141	0.2924	Arg	CGU	578	2.4372
Gly	GGU	1076	2.23	Ser	AGC	72	0.2292
His	CAC	87	0.3858	Ser	AGU	512	1.6278
His	CAU	364	1.6142	Ser	UCA	716	2.2764
Ile	AUA	264	0.2976	Ser	UCC	61	0.1938
Ile	AUC	281	0.3168	Ser	UCG	93	0.2958
Ile	AUU	2116	2.3856	Ser	UCU	433	1.377
Lys	AAA	1899	1.8356	Thr	ACA	897	2.0296
Lys	AAG	170	0.1644	Thr	ACC	104	0.2352
Leu	CUA	213	0.399	Thr	ACG	158	0.3576
Leu	CUC	21	0.0396	Thr	ACU	609	1.378
Leu	CUG	37	0.069	Val	GUA	615	1.412
Leu	CUU	346	0.648	Val	GUC	111	0.2548
Leu	UUA	2430	4.5492	Val	GUG	104	0.2388
Leu	UUG	158	0.2958	Val	GUU	912	2.094
Met	AUA	2	0.0245	Trp	UGG	350	1
Met	AUC	0	0	Tyr	UAC	189	0.378
Met	AUG	560	6.8775	Tyr	UAU	811	1.622

Table S6. The nucleotide variability of *N. incert* chloroplast genome.

Number	Region	Pi	Total Number of mutations	Region length
1	IR.gene1.psbY	0.13889	27	111
2	IR.gene2.rrn5	0.04795	13	137
3	IR.gene3.rrn23	0.02968	182	2889
4	IR.gene4.trnA-UGC	0.03196	5	74
5	IR.gene5.trnI-GAU	0.00901	2	74
6	IR.gene6.rrn16	0.02121	69	1487
7	IR.gene7.ycf89	0.216	410	1086
8	IR.gene8.trnP-UGG	0.01826	4	76
9	LSC.gene1.ycf45	0.20146	511	1383
10	LSC.gene10.petD	0.14037	138	483
11	LSC.gene11.petB	0.1286	166	675
12	LSC.gene12.trnS-UGA	0.01804	4	88
13	LSC.gene13.psaD	0.13587	117	420
14	LSC.gene14.trnM-CAU	0.00694	1	76
15	LSC.gene15.rpl12	0.17882	141	387
16	LSC.gene16.rpl1	0.20763	289	693
17	LSC.gene17.rpl11	0.15149	137	426
18	LSC.gene18.trnW-CCA	0.02441	3	75
19	LSC.gene19.dnaB	0.27662	695	1383
20	LSC.gene2.rpl20	0.1821	126	345
21	LSC.gene20.trnF-GAA	0	0	73
22	LSC.gene21.psbX	0.18917	43	174
23	LSC.gene22.ycf66	0.17646	103	309
24	LSC.gene23.psbV	0.16233	164	492
25	LSC.gene24.trnR-CCG	0.01967	3	73
26	LSC.gene25.trnM-CAU	0.02857	5	87
27	LSC.gene26.rpl19	0.21889	154	447
28	LSC.gene27.petF	0.12644	80	300
29	LSC.gene28.petA	0.15591	301	945
30	LSC.gene29.tatC	0.15184	218	741
31	LSC.gene3.rpl35	0.1894	74	195
32	LSC.gene30.atpE	0.12687	105	402
33	LSC.gene31.atpB	0.11092	327	1428
34	LSC.gene32.ycf3	0.13056	133	534
35	LSC.gene33.rps18	0.16307	71	219
36	LSC.gene34.rpl33	0.13299	54	195
37	LSC.gene35.rps20	0.23239	130	282
38	LSC.gene36.rpoB	0.19809	1632	4219
39	LSC.gene37.rpoC1	0.1831	645	2136
40	LSC.gene38.rpoC2	0.23487	1897	4290
41	LSC.gene39.rps2	0.15382	219	690
42	LSC.gene4.psaE	0.17847	75	201
43	LSC.gene40.atpA	0.12394	390	1518
44	LSC.gene41.atpD	0.15567	186	564
45	LSC.gene42.atpF	0.14346	161	543
46	LSC.gene43.atpG	0.13461	136	471

47	LSC.gene44.atpH	0.0656	37	249
48	LSC.gene45.atpI	0.12638	189	729
49	LSC.gene46.sufC	0.17778	272	765
50	LSC.gene47.sufB	0.15829	460	1461
51	LSC.gene48.rbcL	0.07486	233	1473
52	LSC.gene49.rbcS	0.11667	102	423
53	LSC.gene5.ftsH	0.16368	609	1881
54	LSC.gene50.psbB	0.09499	314	1530
55	LSC.gene51.psbT	0.10764	21	99
56	LSC.gene52.psbN	0.11717	29	132
57	LSC.gene53.psbH	0.09837	47	204
58	LSC.gene54.petN	0.08963	19	90
59	LSC.gene55.petM	0.16848	46	129
60	LSC.gene56.trnD-GUC	0	0	76
61	LSC.gene57.trnS-GCU	0.00397	1	89
62	LSC.gene58.trnM-CAU	0.00926	2	73
63	LSC.gene59.ycf33	0.30256	108	195
64	LSC.gene6.rps14	0.19142	116	303
65	LSC.gene60.trnY-GUA	0.03902	7	84
66	LSC.gene61.trnV-UAC	0	0	74
67	LSC.gene62.trnR-UCU	0.03192	6	74
68	LSC.gene63.trnT-UGU	0.00926	2	72
69	LSC.gene64.petG	0.11287	26	114
70	LSC.gene65.psbK	0.10914	32	135
71	LSC.gene66.psaI	0.12132	29	111
72	LSC.gene67.psbD	0.07462	169	1056
73	LSC.gene68.psbC	0.09336	280	1446
74	LSC.gene69.trnK-UUU	0.03056	6	72
75	LSC.gene7.psaM	0.12975	26	93
76	LSC.gene70.ycf12	0.09333	20	105
77	LSC.gene71.psbZ	0.12401	50	186
78	LSC.gene72.trnG-GCC	0	0	75
79	LSC.gene73.trnE-UUC	0.00913	2	75
80	LSC.gene74.ycf90	0.23215	568	1296
81	LSC.gene75.psbJ	0.11667	29	120
82	LSC.gene76.psbL	0.06752	15	117
83	LSC.gene77.psbF	0.10707	30	132
84	LSC.gene78.psbE	0.05203	31	255
85	LSC.gene79.trnG-UCC	0	0	71
86	LSC.gene8.chlI	0.15247	320	1062
87	LSC.gene80.ycf4	0.18083	193	546
88	LSC.gene81.petL	0.14097	31	96
89	LSC.gene82.psaL	0.14243	133	456
90	LSC.gene83.cbbX	0.14792	258	873
91	LSC.gene84.ycf39	0.17833	348	960
92	LSC.gene85.ycf41	0.36394	213	354
93	LSC.gene86.psbI	0.08632	21	117
94	LSC.gene87.psaF	0.17885	198	558

95	LSC.gene88.psaJ	0.14974	37	126
96	LSC.gene89.psaA	0.10707	517	2259
97	LSC.gene9.secG	0.24509	104	219
98	LSC.gene90.psaB	0.10996	506	2322
99	SSC.gene1.rpl32	0.20121	67	180
100	SSC.gene10.psbA	0.05294	122	1083
101	SSC.gene11.rps16	0.22963	110	240
102	SSC.gene12.rps4	0.19642	244	618
103	SSC.gene13.trnH-GUG	0.01644	3	75
104	SSC.gene14.syfB	0.30985	1228	2136
105	SSC.gene15.psb28	0.14693	105	348
106	SSC.gene16.trnQ-UUG	0.01667	3	74
107	SSC.gene17.trnR-ACG	0.02922	4	76
108	SSC.gene18.groEL	0.16	503	1602
109	SSC.gene19.dnaK	0.16198	603	1842
110	SSC.gene2.trnL-UAG	0.03951	9	82
111	SSC.gene20.rpl3	0.16592	209	639
112	SSC.gene21.rpl4	0.19187	248	648
113	SSC.gene22.rpl23	0.1644	103	312
114	SSC.gene23.rpl2	0.16655	279	828
115	SSC.gene24.rps19	0.16882	89	288
116	SSC.gene25.ycf88	0.25497	235	489
117	SSC.gene26.rpl22	0.14023	103	348
118	SSC.gene27.rps3	0.15969	200	645
119	SSC.gene28.rpl16	0.14245	118	417
120	SSC.gene29.rpl29	0.1722	76	234
121	SSC.gene3.rbcR	0.19098	351	924
122	SSC.gene30.rps17	0.13281	76	270
123	SSC.gene31.rpl14	0.14699	107	366
124	SSC.gene32.rpl24	0.17389	85	249
125	SSC.gene33.rpl5	0.17843	263	723
126	SSC.gene34.rps8	0.18453	135	399
127	SSC.gene35.rpl6	0.23364	238	588
128	SSC.gene36.rpl18	0.18578	153	408
129	SSC.gene37.rps5	0.16757	169	537
130	SSC.gene38.secY	0.21023	494	1293
131	SSC.gene39.rpl36	0.13509	31	114
132	SSC.gene4.rpl21	0.15304	104	318
133	SSC.gene40.rps13	0.19819	153	372
134	SSC.gene41.rps11	0.16193	121	393
135	SSC.gene42.rpoA	0.17642	339	936
136	SSC.gene43.rpl13	0.25604	231	480
137	SSC.gene44.rps9	0.21399	175	417
138	SSC.gene45.rpl31	0.17966	77	328
139	SSC.gene46.rps12	0.14278	107	381
140	SSC.gene47.rps7	0.1431	139	471
141	SSC.gene48.tufA	0.13745	337	1233
142	SSC.gene49.rps10	0.1786	123	324

143	SSC.gene5.rpl27	0.17671	90	255
144	SSC.gene50.ycf35	0.1863	134	405
145	SSC.gene51.trnL-UAA	0.02588	5	88
146	SSC.gene52.trnC-GCA	0.04507	6	74
147	SSC.gene53.clpC	0.15441	841	2859
148	SSC.gene54.ccsA	0.16315	303	939
149	SSC.gene55.rps6	0.14949	93	297
150	SSC.gene56.trnN-GUU	0.01204	2	72
151	SSC.gene57.psaC	0.1168	56	246
152	SSC.gene6.secA	0.18297	971	2661
153	SSC.gene7.rpl34	0.21905	65	147
154	SSC.gene8.ycf46	0.16444	487	1494
155	SSC.gene9.ccs1	0.20769	512	1266

1

Table S7. Comparison information of chloroplast and mitochondrial genome in *N. incerta.*

query-chl	subject-mt	percentage of identical matches	length	number of mismatches	number of gap openings	start of alignment in query	end of alignment in query	start of alignment in subject	end of alignment in subject	expect value	bitscore	gene (cp)	gene (mt)
Chloroplast	1	74.275	276	66	5	73543	73815	42815	42542	1.61e-24	111	rrn23 (partical:9.87%)	rrl (partical: 2.73%)
Chloroplast	1	74.275	276	66	5	122983	123255	42542	42815	1.61e-24	111	rrn23 (partical:9.87%)	rrl (partical: 2.73%)
Chloroplast	1	81.633	98	18	0	72944	73041	48235	48138	1.26e-15	82.4	rrn23 (partical:3.54%)	rrl (partical: 0.98%)
Chloroplast	1	81.633	98	18	0	123757	123854	48138	48235	1.26e-15	82.4	rrn23 (partical:3.54%)	rrl (partical: 0.98%)

Table S8. Transferred genes between the mtDNA and cpDNA of *N. incerta*.

query-chl-pep	subject-mt	percentage of identical matches	length	number of mismatches	number of gap openings	start of alignment in query	end of alignment in query	start of alignment in subject	end of alignment in subject	expect value	bitscore
petB_len216	1	38.462	182	111	1	34	215	52988	52446	2.83e-36	127
petD_len161	1	44.578	83	42	2	65	144	52301	52056	3.34e-16	67
rpl14_len122	1	35.433	127	77	2	1	122	10764	10384	2.59e-24	88.6
rpl16_len139	1	43.066	137	76	1	4	138	4187	3777	9.28e-34	116
rpl2_len275	1	53.968	189	82	4	65	252	6177	5623	3.18e-56	186
rpl5_len239	1	30.081	123	80	2	40	160	10312	9956	2.30e-07	42.7
rpl6_len196	1	34.444	180	105	4	26	192	14229	13690	2.35e-21	83.2
rps12_len127	1	60	120	48	0	1	120	11685	11326	7.40e-44	144
rps13_len124	1	39.091	110	67	0	1	110	34059	33730	7.51e-16	64.3
rps14_len101	1	30.097	103	64	2	3	101	29511	29215	5.41e-07	38.1
rps19_len94	1	43.038	79	40	2	1	79	5559	5338	1.72e-13	56.2
rps3_len215	1	38.144	97	53	3	117	206	4509	4219	7.82e-12	55.8
rps8_len133	1	29.365	126	86	1	1	126	14647	14279	9.64e-13	55.8
